# Complement inhibition by *Sarcoptes scabiei* protects *Streptococcus pyogenes - *An *in vitro* study to unravel the molecular mechanisms behind the poorly understood predilection of *S*. *pyogenes* to infect mite-induced skin lesions

**DOI:** 10.1371/journal.pntd.0005437

**Published:** 2017-03-09

**Authors:** Pearl M. Swe, Lindsay D. Christian, Hieng C. Lu, Kadaba S. Sriprakash, Katja Fischer

**Affiliations:** QIMR Berghofer Medical Research Institute, Infectious Diseases Department, Herston, Brisbane, Australia; Johns Hopkins Bloomberg School of Public Health, UNITED STATES

## Abstract

**Background:**

On a global scale scabies is one of the most common dermatological conditions, imposing a considerable economic burden on individuals, communities and health systems. There is substantial epidemiological evidence that in tropical regions scabies is often causing pyoderma and subsequently serious illness due to invasion by opportunistic bacteria. The health burden due to complicated scabies causing cellulitis, bacteraemia and sepsis, heart and kidney diseases in resource-poor communities is extreme. Co-infections of group A streptococcus (GAS) and scabies mites is a common phenomenon in the tropics. Both pathogens produce multiple complement inhibitors to overcome the host innate defence. We investigated the relative role of classical (CP), lectin (LP) and alternative pathways (AP) towards a pyodermic GAS isolate 88/30 in the presence of a scabies mite complement inhibitor, SMSB4.

**Methodology/Principal findings:**

Opsonophagocytosis assays in fresh blood showed baseline immunity towards GAS. The role of innate immunity was investigated by deposition of the first complement components of each pathway, specifically C1q, FB and MBL from normal human serum on GAS. C1q deposition was the highest followed by FB deposition while MBL deposition was undetectable, suggesting that CP and AP may be mainly activated by GAS. We confirmed this result using sera depleted of either C1q or FB, and serum deficient in MBL. Recombinant SMSB4 was produced and purified from *Pichia pastoris*. SMSB4 reduced the baseline immunity against GAS by decreasing the formation of CP- and AP-C3 convertases, subsequently affecting opsonisation and the release of anaphylatoxin.

**Conclusions/Significance:**

Our results indicate that the complement-inhibitory function of SMSB4 promotes the survival of GAS *in vitro* and inferably in the microenvironment of the mite-infested skin. Understanding the tripartite interactions between host, parasite and microbial pathogens at a molecular level may serve as a basis to develop improved intervention strategies targeting scabies and associated bacterial infections.

## Author summary

The molecular mechanisms that underpin the link between scabies and bacterial pathogens were unknown. We proposed that scabies mites play a role in the establishment, proliferation and transmission of opportunistic pathogens. We investigated here the synergy between mites and one of the most recognised mite associated pathogens, *Streptococcus pyogenes*. As part of the innate immune response mammals have a pre-programmed ability to recognise and immediately act against substances derived from fungal and bacterial microorganisms. This is mediated through a sequential biochemical cascade involving over 30 different proteins (complement system) which as a result of signal amplification triggers a rapid killing response. The complement cascade produces peptides that attract immune cells, increases vascular permeability, coats (opsonises) the surfaces of a pathogen, marking it for destruction, and directly disrupts foreign plasma membranes. To prevent complement mediated damage of their gut cells, scabies mites secrete several classes of complement inhibiting proteins into the mite gut and excrete them into the epidermal mite burrows. Furthermore, these inhibitors also provide protection for *S*. *pyogenes*. We verified here specifically the impact of the mite complement inhibitor SMSB4, to identify the molecular mechanisms behind the long recognised tendency of *S*. *pyogenes* to infect mite-induced skin lesions.

## Introduction

*Streptococcus pyogenes* or group A streptococcus (GAS) is a human specific pathogen, which can cause a wide variety of diseases that typically originate from localised infections of skin (impetigo) or throat (pharyngitis). Multiplication and lateral spread of GAS invading the skin can result in erysipelas and cellulitis in the deep layers of the skin or in necrotising fasciitis. Disease progression from here can cause severe systemic infections such as streptococcal toxic shock syndrome (STSS) and life-threatening sepsis. Autoimmune-mediated complications, in particular, rheumatic heart disease (RHD) and post-streptococcal glomerulonephritis (PSGN) can develop after the initial infection has resolved. To date, GAS remains in the top ten global causes of mortality with at least approximately 500,000 deaths a year [1, 2].

Scabies, caused by infection with *Sarcoptes scabiei*, is an important risk factor for impetigo resulting from GAS and *Staphylococcus aureu*s infections [2–6]. Inhibition of innate defences including the complement system is a prerequisite for successful establishment of bacterial infections. GAS and *S*. *aureus* have evolved mechanisms to prevent activation of the complement cascades [7–16]. Recently we have shown that scabies mites may offer further congenial conditions for infections by these bacteria by flooding their immediate surroundings with a multitude of complement inhibitors [17–20]. In particular the scabies mite serpin B4 (SMSB4), a 54 kDa serine protease inhibitor, inhibits complement activation [20] and promotes the growth of GAS [19] and *S*. *aureus* [21]. SMSB4 is secreted into the mite digestive system, where it co-localises with ingested host complement factors [20] and it is excreted with the mite faeces into the epidermal mite burrows [20]. Bacteria, in particular cocci, have been found in great abundance in the epidermal mite burrows [22]. In multiple clinical reports the colonisation of mite-infected skin with GAS [23], *S*. *aureus* [22, 24], and other pathogens [25, 26] has been thought to be the main cause of systemic infection and detrimental disease outcomes for patients with severe scabies.

The complement system, an immediate host defence against invading pathogens, consists of more than 30 soluble plasma proteins that constitute a series of enzymatic cascades [27]. Complement can be activated via three different pathways, namely classical pathway (CP), lectin pathway (LP) and alternative pathway (AP). The CP is antibody-dependent and initiated by binding of C1q, a pattern recognition molecule (PRM) to the bacterial bound immune complexes such as IgG, natural IgM or direct binding to surface microbial sugars [28–30]. The LP is initiated when microbial surface sugars are recognised by the PRMs, mannose binding lectin (MBL) or M-,L- and H-ficolins. These two pathways form the enzyme complex CP/LP-C3 convertase (C4b2a) [31–33]. In the AP, C3 naturally breaks down to C3_H2O_ at a low level to which factor B (FB) binds, and this assembly is cleaved by factor D, forming an AP-C3 convertase (C3bBb) [34]. This enzyme complex generally requires stabilisation by properdin [35, 36]. The C3 convertase is the key enzyme resulting from the complement activation, and it cleaves C3 to release an important opsonin, C3b. Deposition of C3b on the microbial surface is crucial as it marks the microbes for an efficient uptake and subsequent killing by phagocytes. Furthermore, at a high local concentration C3b binds to C3 convertase, thereby turning into C5 convertase (C4b2a3b/C3bBb3b). C5 convertase cleaves C5 into C5a and C5b. C5a is a potent chemoattractant, which recruits neutrophils, monocytes and macrophages to the site of infection. C5b and other complement components (C6, C7, C8 and C9) form the membrane attack complex (MAC/C5b-9) on the cell surface, causing direct cell lysis in sensitive cells, such as gram-negative bacteria [37, 38].

To date, studies on interactions between complement and GAS were only focused on the CP and AP [39–42]. Here we investigate the role of all three complement pathways innately controlling establishment of GAS infection. We found that CP plays a major role followed by AP, while the role of MBL-dependent LP was insignificant. Furthermore, we analysed the role of the scabies mite complement inhibitor SMSB4 in the survival of GAS in fresh blood to better understand the mechanisms underlying the link between GAS and scabies when co-infecting the human host. Our data showed that SMSB4 promoted the growth of GAS in blood by inhibiting the activation of the CP and the AP, which presumably caused the reduction of opsonisation and anaphylatoxin release. This is the first study analysing molecular interactions that may govern the initial events of overcoming human complement defence during co-infection of the skin by scabies mites and GAS.

## Methods

### Ethics statement

Normal human serum (NHS) for complement activation assays and fresh blood samples for bactericidal assays were prepared from blood donated by healthy volunteers. Informed written consent was obtained from all blood donors. Blood from one donor was used in all further assays requiring fresh whole blood. The protocols for sourcing blood for complement assays were approved by the Human Research Ethics Committee of the QIMR Berghofer Medical Research Institute (P443).

### Preparation of NHS

Ten ml of venous blood collected into a Vacutainer (Becton Dickinson) was obtained from at least 7 healthy volunteers. Tubes containing the blood samples were allowed to clot at room temperature (RT) for 30 min. Samples were centrifuged at 2000 ×*g* for 10 min at 4°C and the clotted blood was removed. Samples were centrifuged again at 2000 ×*g* for 10 min at 4°C. Sera were pooled, aliquoted into 500 μl volumes and stored at -80°C until use.

### Complement depleted/deficient sera

Depleted sera (C1q^-^ and FB^-^) were purchased from Quidel (San Deigo, USA). These sera were prepared from pooled human sera from healthy donors, which were specifically depleted of either C1q or FB. MBL deficient serum (MBL^d^) was purchased from the Statens Serum Institut (Copenhagen, Denmark). It was prepared from pooled sera from blood collected from otherwise healthy donors with the MBL genotype B/B.

### Removal of IgG from NHS

IgG was depleted from NHS using Albumin and IgG depletion SpinTrap columns prepacked with Protein G Sepharose (GE Healthcare), following the manufacturer’s instructions.

### Bacterial strains and growth conditions

GAS isolates were obtained from the culture collection from the scabies and bacterial pathogenesis laboratory at QIMR Berghofer MRI. Strains used here were GAS 88/30 (*emm* 97) [43, 44], PRS30 (*emm* 83) [45], both *emm*-cluster D, PRS8 (*emm* 12) [45], 5448 (*emm* 1)[46], both *emm*-cluster A-C, PRS55 (*emm* 9), PRS15 (*emm* 48), both *emm*-cluster E [45]. All strains were cultured at 37°C and 5% CO_2_ either on Columbia Blood Agar supplemented with 0.1% CaCO_3_ (w/v) and 4% defibrinated horse Blood (Equicell products, Australia) (CBAC) or in Tryptic Soy Broth (Thermo Fisher Scientific Pty. Ltd., Australia) (TSB).

### Preparation of cell suspensions

*GAS* cell suspensions were prepared from mid-log growth phase cultures (OD_600_ = 0.35). Cells were harvested by centrifugation (4000 ×*g*, 10 min, 4°C), washed twice in phosphate buffered saline (PBS) and re-suspended to a final OD_600_ = 0.03 in the same buffer. This cell suspension corresponds to approximately 1x 10^5^ colony forming units (cfu)/ml. Bacteria were enumerated by plate count of cfu/ml on CBAC agar at 37°C and 5% CO_2_ overnight.

### Production and purification of recombinant SMSB4

DNA encoding SMSB4 was cloned and expressed in *Escherichia coli* BL21 (Qiagen), purified under denaturing condition and refolded into active serpin as described previously [21]. Briefly, SMSB4 cDNA (Yv5004A04, GenBank accession no. JF317222) of the human scabies mite *S*. *scabiei* cloned into the pQE9 expression vector (Qiagen) was transformed into *E*. *coli* BL21. *E*. *coli* cells were cultivated overnight at 37°C in Luria broth (Becton Dickinson) containing 100 μg/mL ampicillin. After inoculation in 2YT medium (Becton Dickinson) containing 100 μg/mL ampicillin, the cells were grown at 37°C, shaking at 200 rpm until an OD_600_ of 0.6–0.7 was reached. Expression of recombinant SMSB4 was induced by addition of 0.5 mM IPTG and continued shaking at 200 rpm for a further 4 h. Cells were collected by centrifugation at 6000 ×*g* at 4°C for 20 min, re-suspended in serpin buffer (50 mM Tris, pH 8.0, 100 mM NaCl, 10 mM EDTA, 1 mM PMSF) and lysed in 250 μg/ml lysozyme and 10 μg/ml DNase at room temperature (RT) under continuous rotation for 1 h. All of the following purification steps were performed at 4°C. After sonication of the spheroplasts by a Sonifier 250 (Branson), inclusion bodies were washed five times using serpinX buffer (50 mM Tris, pH 8.0, 100 mM NaCl, 10 mM EDTA, 0.5% (v/v) Triton X-100) and retrieved by centrifugation (16,000 ×*g* for 20 min at 4°C). The resulting pellet was dissolved in solubilisation buffer (6 M guanidine hydrochloride, 50 mM Tris, pH 7.8, 1 mM DTT) for 1 h. Proteins were further purified by nickel affinity chromatography. Solubilised protein was diluted 1:1 with bind buffer (6 M urea, 100 mM NaH_2_PO_4_, 10 mM Tris, pH 8.0, 5 mM imidazole, 150 mM NaCl, 1% (v/v) glycerol, 1 mM DTT) and bound overnight to a pre-equilibrated 1 ml Ni-NTA matrix (Qiagen) in a PolyPrep column (BioRad) on a rotating shaker. The column was washed twice with 5 ml of wash buffer (6 M urea, 100 mM NaH_2_PO_4_, 10 mM Tris, pH 6.3, 5 mM imidazole, 150 mM NaCl, 1% (v/v) glycerol, 1 mM DTT). Bound proteins were eluted twice using 3 ml of elution buffer (6 M urea, 100 mM NaH_2_PO_4_, 10 mM Tris, pH 8.0, 250 mM imidazole, 150 mM NaCl, 1% (v/v) glycerol and 1 mM DTT). Purified recombinant proteins were refolded overnight by drop wise addition of the protein elution into refolding buffer (300 mM L-arginine, 50 mM Tris, 50 mM NaCl and 5 mM DTT, pH 10.5) using a Minipuls 3 pump (Gilson) at a flow rate of 20 μl/min under gentle stirring. Refolded proteins were concentrated using an Ultrasette Lab Tangential Flow Device (10 kDa MWCO, PALL Life Sciences), followed by further concentration in centrifugal filters (10 kDa MWCO, Amicon Ultra, Millipore). Protein concentrations were determined by Bradford protein assay (Bio-Rad) with bovine serum albumin (BSA) (Invitrogen) as a standard according to the manufacturer’s instructions. Molecular mass and purity were confirmed using SDS-PAGE analysis with Coomassie blue R-250 staining. For all assays, SMSB4 was buffer exchanged into the corresponding assay buffers using 0.5 ml centrifugal filters (10 kDa MWCO, Amicon Ultra, Millipore).

### Bactericidal assays

Bactericidal assays were performed with fresh human blood collected in standard vacutainers containing hirudin as anticoagulant at a concentration of 25 μg/ml (Dynabyte Informationssysteme GmbH, Munich, Germany). Hirudin (lepirudin) generally preserves the complement reactivity, making it the most suited anticoagulant for complement *in vitro* studies [47, 48]. The assays were performed as described previously [21] with minor modifications. Bacteria were grown overnight at 37°C and 5% CO_2_ in 5 ml TSB. The overnight culture was diluted to an initial OD_600_ of 0.05 in a fresh aliquot of 5 ml TSB and the GAS culture was grown to mid-log growth phase (OD_600_ 0.35) at 37°C and 5% CO_2_. This culture was diluted in PBS to obtain an approximately 1×10^3^ cfu/ml challenge dose. To 100 μl of human venous blood, either of the following compounds were added in a volume of 27.5 μl: purified recombinant SMSB4 in the experimental samples, BSA or GVB^2+^ buffer (5 mM veronal buffer, 140 mM NaCl, 0.1% (w/v) gelatin, 1 mM MgCl_2_, 0.15 mM CaCl_2_, pH 7.35) in the negative controls. Finally 12.5 μl of the GAS suspension were mixed into a total volume of 140 μl. Samples were placed on a rotisserie and incubated with end over end mixing for 3 h at 37°C. Subsequently 50 μl aliquots from each appropriately diluted tube were plated in duplicate on CBAC agar plates. The plates were incubated overnight at 37°C and 5% CO_2_ and bacterial numbers were enumerated as cfu/ml. Bacterial recovery was calculated as a percentage of the number of bacteria recovered from samples treated with various test compounds in reference to the GAS challenge dose in PBS without addition of blood.

### Complement depositions assay on GAS

To coat a 96-well assay plate (Maxisorp Immuno Plate, Nunc, Denmark) with GAS cells, 100 μl of approximately 1×10^5^ cfu/ml of GAS cell suspension was added to the wells, incubated first at 37°C for 1 h and subsequently kept at 4°C overnight. Wells were washed 4 times with 200 μl PBS and 0.05% Tween-20 in between each step of the assay. The cells were incubated with blocking buffer (4% BSA in PBS and 0.05% Tween-20) for 2 h at RT. Meanwhile, aliquots of 35 μl of 10% pooled human serum diluted in GVB^2+^ buffer were incubated with 35 μl of SMSB4 or BSA of varying concentrations at 37°C, 200 rpm for 1 h in a V-shaped bottom 96-well plate (Nunc). Sixty μl of these mixtures were then transferred to the wells of GAS coated plate, which was further incubated at 37°C for 1 h. Bound complement proteins were detected by incubation with 60 μl of primary antibodies against human complement factors for 1 h at RT. For immunodetection, antibodies against C1q, C3d, C4c (Dako, Denmark), properdin (R&D systems), sC5b-9 neoantigen-specific antibody recognising the MAC complex (Complement Technology Inc., USA), IgG (Sigma) were used at a dilution of 1:4000 and antibodies against FB (Complement Technology Inc., USA), MBL, Ficolin H (R&D system), Ficolin M and L (Thermo Scientific) were used at dilution of 1:1000. The wells were subsequently incubated with 60 μl of horseradish peroxidase (HRP)-conjugated goat anti-rabbit, HRP-conjugated rabbit anti-goat, HRP-conjugated goat anti-mouse secondary antibodies (Dako, Denmark) at dilutions of 1:1000–1:4000 in blocking buffer at RT for 30 min to 1 h, depending on the primary antibody specificity and signal intensity. Sixty μl of OPD reagent (Dako, Denmark) containing 0.01% hydrogen peroxide was added to each well and incubated at RT until the ‘serum only’ positive control turned yellow. Reactions were stopped by addition of 50 μl of 0.5 N H_2_SO_4_ and absorbances were measured at OD_490_ with a POLARstar Optima fluorescent microtiter plate reader (BMG Labtech, Melbourne, Australia).

### Statistical analysis

Statistical significance was determined using one way or two way ANOVA, with Tukey’s, Dunnett’s or Sidak’s multiple comparisons tests (GraphPad Prism software, version 6.0; GraphPad Software Inc. USA). Values of p<0.05 were considered significant.

## Results

### Assessment of clinical GAS isolates for baseline immunity

**Fig 1:**
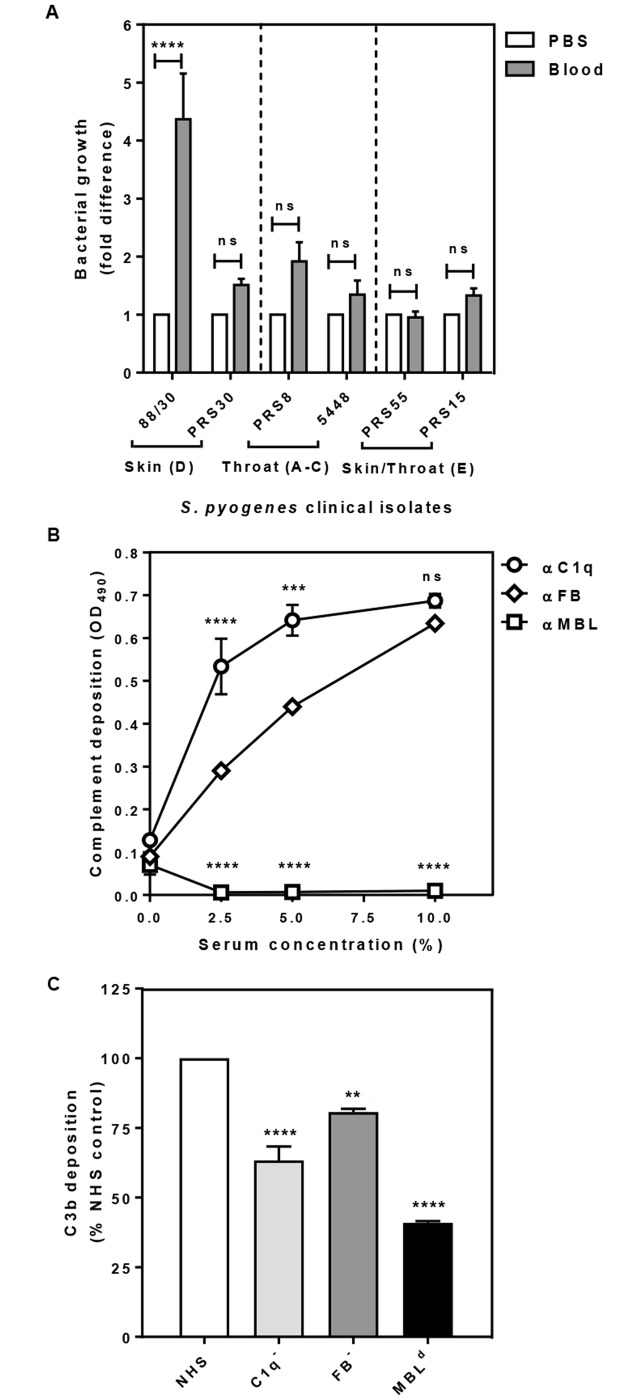
GAS clinical isolates are naturally resistant to blood killing (A) and deposition of C1q, MBL, FB (B) and C3b, indicative of opsonisation (C) on the cell surface of GAS 88/30. Skin strains 88/30, PRS30 (*emm* cluster D), throat strains PRS8, 5448 (*emm* cluster A-C) and skin/throat strains PRS55, PRS15 (*emm* cluster E) were harvested from mid-log growth phase culture (OD_600_ 0.35). Suspension of GAS in PBS (1 ×10^3^ cfu/ml) were added into fresh blood pre-treated with GVB^2+^ buffer. After 3 h incubation samples were plated in duplicate on CBAC agar plates and bacteria were enumerated as cfu/ml. GAS cell in PBS without blood at time 0 (T0) was plated simultaneously and the numbers of bacteria grown served as the baseline for normalisation and for calculating the fold difference of bacteria numbers grown from the experimental samples **(A)**. Maxisorp 96-well plates coated with GAS cells were incubated with increasing concentrations of NHS **(B)** or with 5% NHS or sera depleted of either C1q (C1^-^) or FB (FB^-^) and serum naturally deficient in MBL (MBL^d^) **(C)**. Complement deposition was detected by ELISA using primary human specific antibodies, followed by HRP-conjugated secondary antibodies, and fluorescence was detected at 490 nm **(B, C)**. Data represent the means ± SEM from three independent experiments. The statistical significance of differences between samples was estimated using two way ANOVA with Tukey’s multiple comparison test **(A, B)** and one way ANOVA with Dunnett’s multiple comparison tests **(C)**. **, *p*<0.01; ***, *p*<0.001; ****, *p*<0.0001, ns, not significant.

GAS strains belong to one of three clusters based on the arrangement of genes for cell surface M- or M-like proteins (*emm* clusters). Members of these clusters exhibit tissue tropisms; cluster A-C GAS strains are generally found in the throat while cluster D is skin tropic and cluster E is referred as generalists with no specific tropism [43]. For this study we needed to define the baseline effect of complement on GAS strains belonging to these clusters. Hence, we selected six clinical strains (two from each cluster) and assessed their ability to survive in fresh human blood containing active complement and phagocytes. GAS strains 88/30 [43, 49] and PRS30 [45] belong to *emm* cluster D and are predominantly recovered from skin. PRS8 [45] and 5448 [46] are of *emm* cluster A-C and exhibit throat tropism. PRS55 and PRS15 [45] belong to *emm* cluster E and are generalists (either skin and/or throat tropism). All these strains survived in blood during the 3 h incubation period showing between 1.5- and 5- fold growth relative to the control, which had PBS in place of blood (Fig 1A). These results suggest that irrespective of the differences in preferential tissue tropism, all strains had a similar level of resistance to whole blood. Furthermore, as the strains survived in the blood, the blood samples had little, if any, opsonic antibodies. Nonetheless, we did not find a growth increase of >32 fold, which is expected in these assays with a 3h incubation in blood in the absence of type-specific antibodies [50–53]. Hence, there seemed to be a growth-attenuation in blood, presumably due to the presence of generalised IgGs and active complement. To further characterise this baseline immunity in subsequent experiments, we used the skin-tropic strain 88/30, which showed the highest survival in blood (~5 fold growth).

### The classical and alternative complement pathways are targeting GAS 88/30

We investigated the deposition of complement components on the GAS surface in the initial activation step of the complement cascades, specifically C1q for CP, FB for AP and MBL for LP. The rationale for this experiment was to determine the contribution of each complement pathway by assessing which components are deposited on the surface of GAS. Ninety six-well Maxisorp plates coated with GAS cells were exposed to an increasing concentration of NHS, and deposition of each complement component on the bacteria cell surface was detected by ELISA using complement factor -specific antibodies. While the amount of both C1q and FB deposited on the GAS cell surface was proportional to the concentrations of serum used, MBL did not appear to bind to the GAS cell surface at any of the serum concentrations tested (Fig 1B). This result suggested that CP and AP, but not MBL-dependent LP are activated by GAS. Since the LP could be triggered by other lectin pathway PRMs such as M-, L- and H-ficolins, we investigated the deposition of these molecules onto the cell surface of 88/30. None of the ficolins deposited (supplementary data, S1 Fig), indicating that the LP may not be important for controlling GAS.

**Fig 2:**
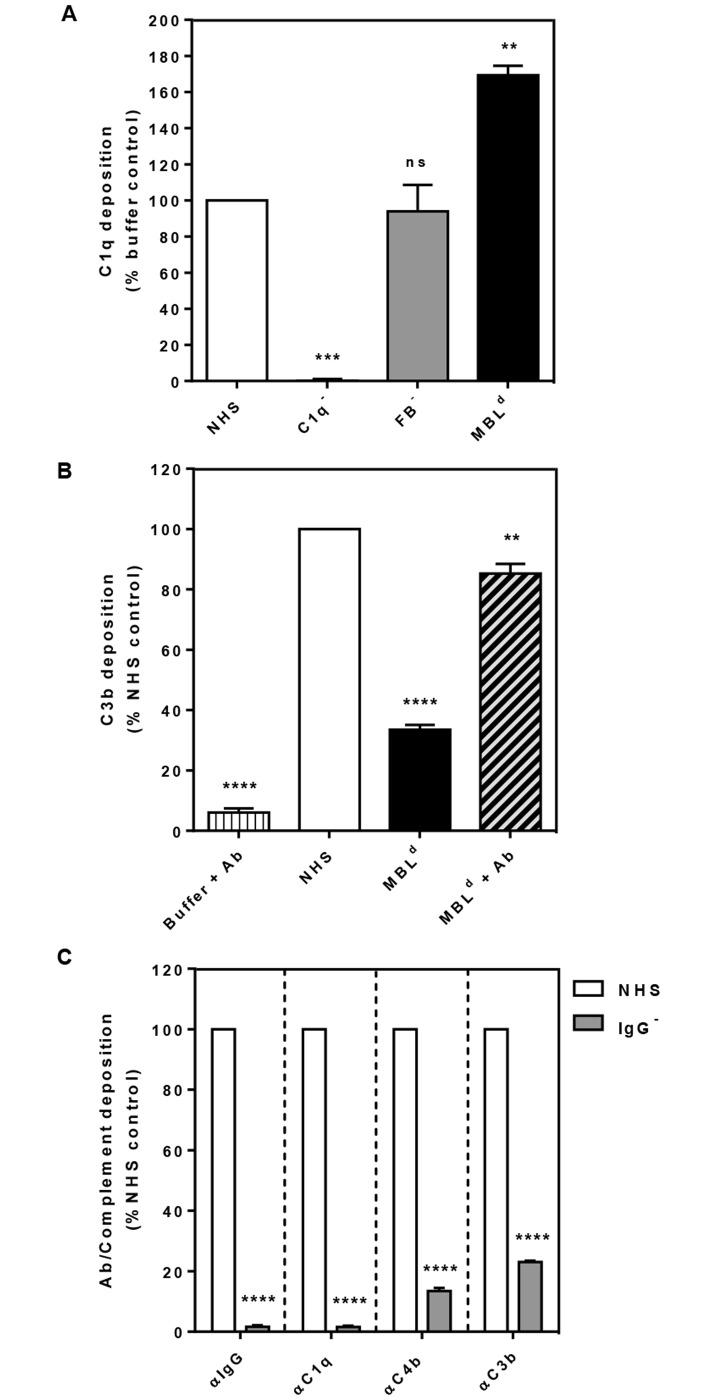
Level of C1q deposition (A), effect of anti-GAS antibodies on C3b deposition (B), and the role of IgG on the CP-dependent deposition of various complement components (C) on the surface of GAS 88/30. Complement deposition was detected by ELISA using primary human-specific antibodies, followed by HRP-conjugated secondary antibodies, and fluorescence was detected at 490 nm. Depositions of IgG and the complement components C1q, C4b and C3b are shown in panels (left to right) in the presence (white column) and absence (grey column) of IgG **(C)**. Results are shown as means ± SEM from three independent experiments. The statistical significance of differences between samples was estimated using one way ANOVA with Dunnett’s multiple comparison tests with a single pooled variance NHS **(A, B)** and two way ANOVA with Sidak’s multiple comparison test **(C)**. **, *p*<0.01; ***, *p*<0.001; ****, *p*<0.0001, ns, not significant.

To confirm this result by an independent experiment, we investigated deposition of the opsonin C3b on the bacterial surface upon activation of the complement pathway with GAS using three commercially sourced human sera, C1 depleted (C1^-^), FB depleted (FB^-^) and MBL deficient (MBL^d^). We expected that the absence of either classical or alternative pathway activation would result in lowered C3b deposition. Accordingly, we found that C3b deposition decreased by 40% in C1^-^ and 20% in FB^-^ sera relative to the NHS control (Fig 1C). This data suggested that both CP, and to a lesser extent AP are responsible for the deposition of C3b on the surface of GAS in this assay. We also observed a 60% reduction in C3b deposition with the MBL^d^ serum (Fig 1C). To address this result obtained from the MBL^d^ serum, we compared all three sera in a C1q deposition assay (Fig 2A). As expected, the C1q^-^ and FB^-^ sera showed respectively little and normal levels of C1q deposition. By contrast, the MBL^d^ serum showed 50–75% more C1q deposition compared to NHS (*p*<0.0001). From these results we inferred that the baseline level anti-GAS antibodies in MBL^d^ serum may be low. Indeed addition of anti-GAS antibodies to the assay rescued C3b deposition by the MBL^d^ serum (Fig 2B). Thus the reduction observed in the C3b deposition in MBL^d^ serum (Fig 1C) was due to the absence of anti-GAS antibodies required for the activation of CP in this serum.

To confirm that the baseline immunity to GAS was due to the presence of general IgG, we depleted IgG from NHS by protein G sepharose column based affinity chromatography. The IgG depleted serum (99%) reduced the deposition of C1q (99%), C4b (a part of C4b2a, i.e. the CP-C3 convertase) (86%) and C3b (78%) on GAS 88/30 (Fig 2C). Reduction in the deposition of these complement components were directly attributable to the absence of antibody and thus related to the classical pathway. These results concur with the phagocytic killing assays described above (Fig 1A). Taken together, the data supports the conclusion that CP and AP are the main complement pathways in controlling the growth of GAS and that the NHS used had a baseline immunity to GAS, impedimental to the establishment of infection.

### Scabies mite complement inhibitor annuls the baseline immunity

**Fig 3:**
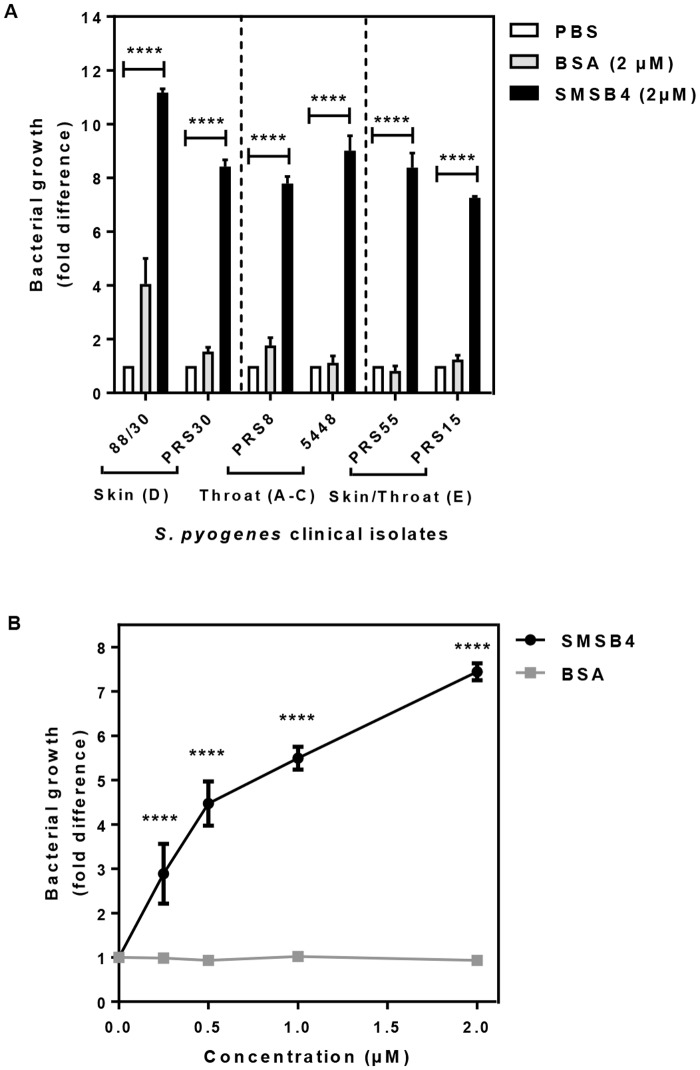
Scabies mite complement inhibitor SMSB4 reduces the baseline immunity of GAS clinical isolates in fresh blood (A) and promotes the growth of GAS skin strain 88/30 in a dose-dependent manner (B). Skin strains 88/30, PRS30 (*emm* cluster D), throat strains PRS8, 5448 (*emm* cluster A-C) and skin/throat strains PRS55, PRS15 (*emm* cluster E) **(A)** or GAS 88/30 only **(B)** were harvested from mid-log growth phase culture (OD_600_ 0.35). GAS diluted in PBS (1 ×10^3^ cfu/ml) were added into fresh blood pre-treated with either 2 μM **(A)** or a range of concentrations **(B)** of either SMSB4 or BSA. After 3 h incubation samples were plated in duplicate on CBAC agar plates and bacteria were enumerated as cfu/ml. The challenge dose of GAS cells in PBS without blood **(A)** or the challenge dose in blood with GVB^2+^ buffer **(B)** was plated simultaneously and the numbers of bacteria grown served as the baseline for normalisation and for calculating the fold difference of bacteria numbers from the experimental samples. Data represent the means ± SEM from three independent experiments. The statistical significance of differences between samples was estimated using two way ANOVA with Tukey’s **(A)** or Sidak’s **(B)** multiple comparison test. **, *p*<0.01; ***, *p*<0.001; ****, *p*<0.0001.

Since we earlier showed that scabies mite SMSB4 is a complement inhibitor and promoted growth of GAS [19], we sought to ascertain whether this protein is able to impact on the growth attenuation of the six diverse strains owing to baseline immunity. We treated blood with 2 μM of recombinant SMSB4 prior to the addition of GAS. This resulted in a significant increase in the numbers of cfu of all strains tested (7–11 fold rise) compared to that of the challenge dose (Fig 3A). We found that SMSB4 promoted growth of 88/30 in a concentration dependent manner (Fig 3B), suggesting that complement could play a significant role in delaying the onset of GAS skin infection.

**Fig 4:**
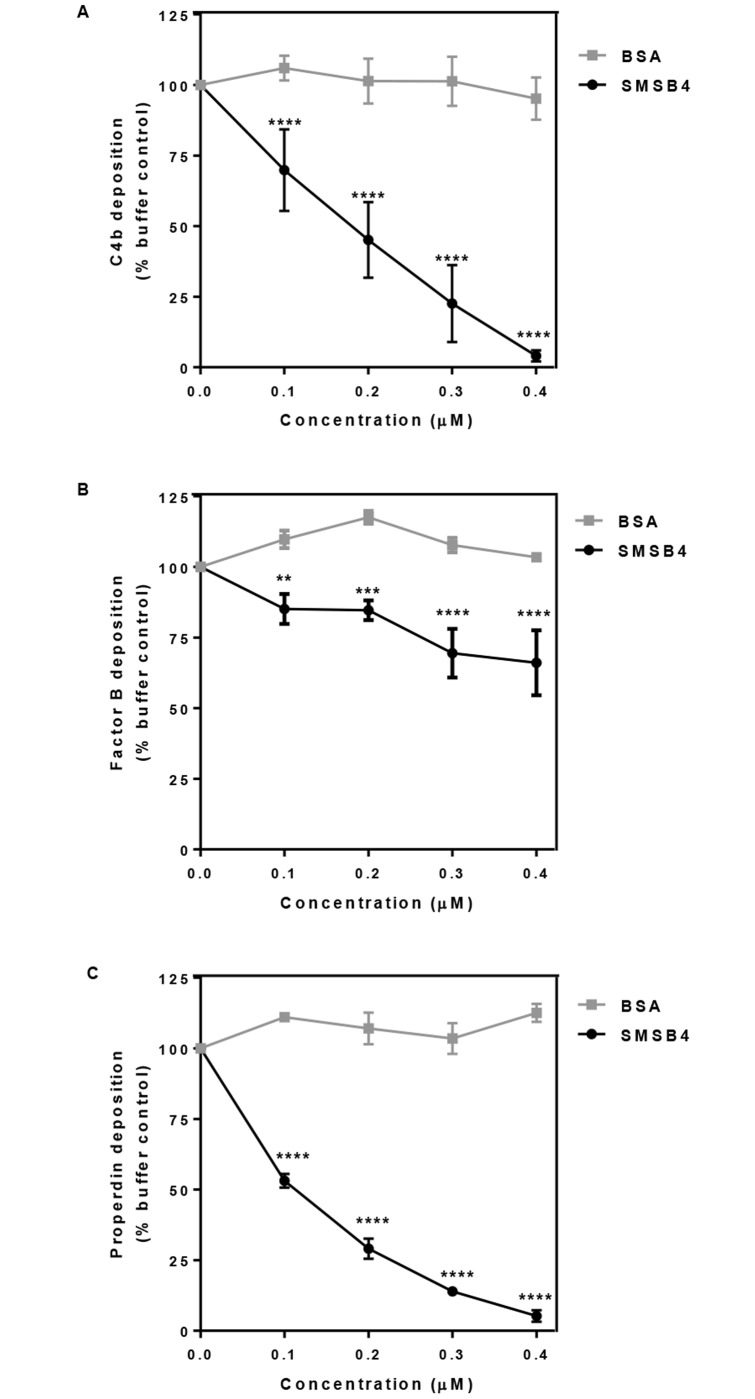
SMSB4 causes reduction in C4b deposition (A) factor B deposition (B) and properdin deposition (C) on GAS 88/30. Cells were incubated with 5% NHS which has been pre-treated with increasing concentrations of either SMSB4 or BSA. Complement deposition was detected by ELISA using primary human specific antibodies, followed by HRP-conjugated secondary antibodies, and fluorescence was detected at 490 nm. Results are shown as means ± SEM from three independent experiments. The statistical significance of differences between BSA and SMSB4 treated samples were estimated using two way ANOVA with Sidak’s multiple comparison test. **, *p*<0.01; ***, *p*<0.001, ****, *p*<0.0001.

To understand SMSB4 mediated inhibition of opsonophagocytosis in blood we investigated the effect of this protein on the formation of CP-C3 convertase (C4b2a), which was measured by deposition of C4b on the 88/30 cells. We demonstrated that SMSB4 caused near complete inhibition of C4b depositions on GAS surface at 0.4 μM concentration (Fig 4A). Likewise, the C3 convertase specifically formed via AP was assayed by probing for the depositions of FB (C3bBb) and properdin (a stabiliser of the AP-C3 convertase). SMSB4 at 0.4 μM concentration caused approximately 25% reduction of FB deposition and near complete reduction of properdin deposition on the GAS surface (Fig 4B and 4C). These results suggest that SMSB4 greatly decreased the formation of CP-C3 convertase, and moderately decreased the formation of AP-C3 convertase.

**Fig 5:**
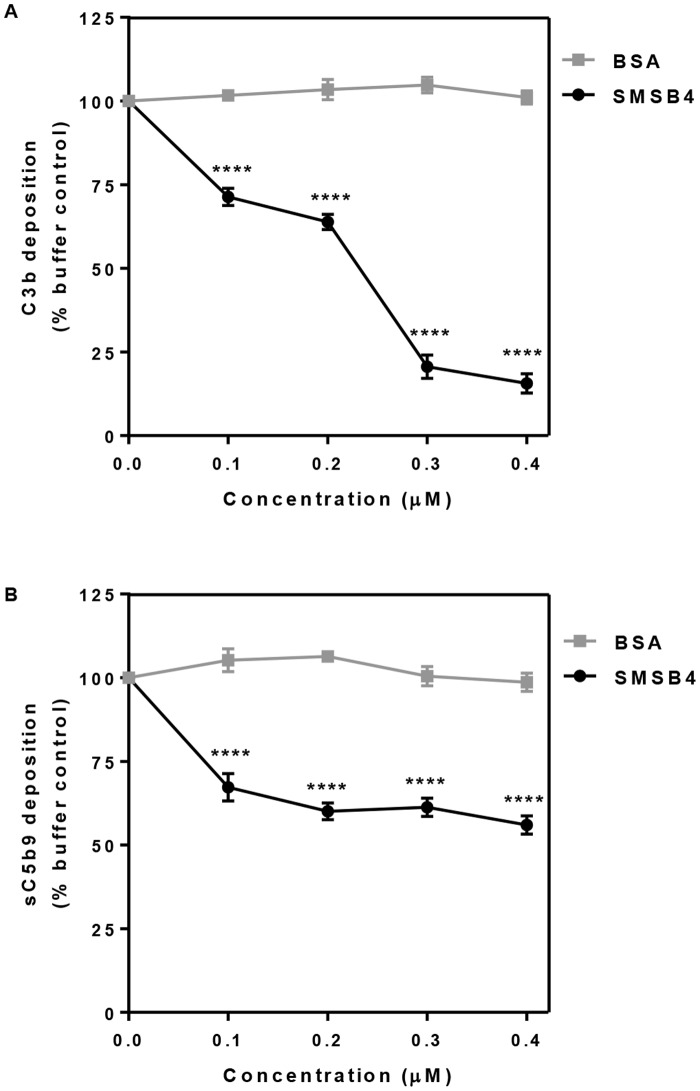
Effect of SMSB4 on opsonisation measured as C3b deposition (A) and the release of anaphylatoxin measured indirectly by ELISA as deposition of sC5b9 complex (B) on GAS 88/30. Cells were incubated with 5% NHS which has been pre-treated with increasing concentrations of either SMSB4 or BSA. Complement deposition was detected by ELISA using primary human specific antibodies, followed by HRP-conjugated secondary antibodies, and fluorescence was detected at 490 nm. Results are shown as means ± SEM from three independent experiments. The statistical significance of differences between BSA and SMSB4 treated samples were estimated using two way ANOVA with Sidak’s multiple comparison test. **, *p*<0.01; ***, *p*<0.001, ****, *p*<0.0001.

Furthermore we tested the effect of SMSB4 on the integral parts of the activated complement network, namely C3b deposition and release of anaphylatoxin C5a. Approximately 85% decrease in C3b deposition was observed when NHS was treated with 0.3 μM SMSB4 (Fig 5A). SMSB4 reduced the deposition of C5b-9 complex by almost 40% when a concentration range of 0.1–0.4 μM was tested (Fig 5B). This was an indirect indication that a proportional reduction in the release of anaphylatoxin C5a occurred in the presence of the mite complement inhibitor SMSB4, as previously observed in a related experiment [54]. Taken together these results illustrated that SMSB4 offers an advantage to GAS by preventing activation of the complement cascade.

## Discussion

The majority of studies regarding host-pathogen interactions of GAS have focused on GAS virulence. Research addressing the relative importance of the host complement response to GAS infection has lagged behind, mainly due to a lack of suitable systems to study such complex interactions. Despite this, it has been established based on a limited number of publications, that CP and AP are major players in controlling GAS infections [39, 41, 42, 55, 56]. However, to our knowledge, a potential role of the LP during GAS infections has not been investigated. There are multiple animal models for analysis of *S*. *pyogenes* pathogenesis [57]. A mouse model genetically engineered to be deficient in specific complement components has been utilised to address the host complement response towards GAS infections [42]. Such models are useful because immune naive serum can be generated from mice and complex interactions between the three complement pathways can be dissected out. However, GAS is not a natural pathogen of mice and considerable differences exist between the murine and human complement systems [16, 58]. Human complement is commonly investigated using human sera, either from donors with normal complement from which specific complement components have been artificially removed, or from patients with a natural deficiency in a specific complement component, e.g. MBL. However, it can be difficult to directly compare results between these commercial sera because the precise composition of individual complement samples cannot be standardised.

Our data is in agreement with previous reports that GAS activates complement via CP and AP [39, 41, 42, 55, 56]. The lack of depositions of MBL and other PRMs of LP on GAS from this study is also in agreement with a previous report by Nordenfelt *et al*. that GAS in human serum were coated with complement proteins of the CP and AP, but not LP [59]. However, the question of whether CP or AP is more important for the activation of complement by GAS remains enigmatic. Initial reports in 1979 using human sera stated that the AP is the primary complement pathway activated in the absence of type-specific IgG [39, 60]. Later work by Carlsson *et al*. in 2003 and 2005 reported that the CP is the main complement pathway in human serum when activated by an M-protein deficient GAS strain [41, 56]. The level of CP-dependent opsonisation may thus depend on the absence or presence of M-protein, as most M-proteins recruit host C4BP to degrade CP-C3 convertase while some M-proteins recruit host factor H (FH), which degrades AP-C3 convertase. More recently in 2006, Yuste *et al*. investigated the host complement response towards four clinical isolates of GAS in mice, genetically engineered to lack either C1q or FB [42]. They compared mice sera with commercial human sera, which were depleted in either C1q or FB. The authors reported that the AP was the main complement pathway activated, mainly because opsonisation and mice survival was most reduced in FB^-^ mice when infected with GAS. It is apparent that there were differences in the complement responses between mice and human sera, and that further differences may be GAS strain-related.

In the particular context of GAS and scabies mite co-infection and the host complement response, the IgG-dependent CP appears to be the predominant complement pathway, in conjunction with a lesser effect of the AP. The GAS strain 88/30 is a skin isolate from a scabies patient (as recorded in the culture collection at the Menzies School of Health Research, Darwin). It carries *emm97*, M protein, which is currently M protein non-serotypable [49]. It is not known whether surface M proteins of 88/30 recruit host regulators such as C4BP and FH [40, 61], which affect the type of complement response [16, 42, 55, 56, 62]. CP-dependent opsonisation would be more important if the GAS surface binds FH, whereas the AP effect may be more pronounced for a strain that recruits C4BP.

Overcoming host innate immunity by GAS is a prerequisite for successful infection. This fine balance between the host and the pathogen may often be influenced by a co-infecting pathogen. In this regard we reported earlier that scabies mites aid GAS infection, presumably by secreting into their immediate surroundings proteins that inhibit complement function [19]. The mechanism or the complement pathways involved were not known. In this study, we described that both CP and AP play a role in the initial immunity against GAS. Furthermore we demonstrate that the scabies mite protein SMSB4 inhibits the formation of C3 convertase mainly via the CP, as indicated by its potent inhibition on C4b deposition. This leads to a reduced opsonisation, which has several downstream effects: a moderate reduction in the AP-C3 convertase, as C3b is a component of this C3 convertase, and a reduced amount of CP- and AP-C3 convertases in combination with decreased C3b deposition, which reduces the amount of C5 convertase formed, and hence impacts on the release of anaphylatoxin C5a. In summary, the scabies mite protein SMSB4 inhibits (i) the formation of C3 convertases via CP and AP, (ii) the deposition of C3b and (iii) the formation of anaphylatoxin C5a. C3b deposition is crucial for the eradication of microbes as it marks the microbes for an efficient uptake and subsequent killing by phagocytes. C5a is a potent chemoattractant, which recruits neutrophils, monocytes and macrophages to the site of infection. We have recently shown that SMSB4 interferes with phagocytosis of *S*. *aureus* by neutrophils [21] and this may also apply to the uptake of GAS. In this light the protection mediated by a recent and highly promising combinatorial synthetic peptide vaccine strategy against GAS [44] may be compromised in scabies patients, as this vaccine critically depends on the presence and action of neutrophils.

Complement is a superabundant system, involving a large number of components in a complex network and among individuals variations in the composition are often observed. Pathogens susceptible to complement have accordingly evolved multiple evasion strategies in order to ensure an anti-complement milieu in their immediate environment. Many pathogens have numerous different complement inhibitors acting on different targets within the complement system. Staphylococci, for example, have evolved an arsenal of molecules to counteract the complement system encoded by about 2% of their total genome [63]. Redundancy is very common and seems to be needed. In this light, it is a captivating thought that pathogens may ‘join forces’ against the onslaught of the host complement defense. Other studies have proposed similar hypotheses, so for example for cysteine proteinases from *Porphyromonas gingivalis* which have been shown to provide an advantage to other periodontal pathogens residing in the same location [64].

Scabies mites have evolved an astonishing repertoire of complement inhibitors, comprised of at least two classes—Scabies Mite Serpins (SMSs) [20] and Scabies Mite Inactivated Serine Proteases (SMIPP-Ss) [65]. These are represented in the genome as multi-copy families. Thirty-three SMIPP-Ss and six serpins have been identified and it is possible that the ongoing mite genome project will identify more. The apparent range of specific mechanisms preventing complement function indicates that mites inhibit the complement system at many points and additive effects of mite complement inhibitors have been demonstrated [20]. Notably, complement factors are ingested by mites [18, 66] but MAC formation is not detected in the gut [66], suggesting that the anti-complement defence system generated by the mite may be indeed very efficient *in vivo*.

The mite serpin SMSB4 is only one of many mite complement inhibitors that scabies mites release simultaneously into the epidermis. We propose that as a whole, mite complement inhibitors accumulate to high anti-complement activities in the confined space of the gut and epidermal burrows, allowing the parasites and associated bacteria to evade the adverse effects of complement activation. This promotes the growth of bacteria present in the confined microenvironment of the epidermal burrows. We do not think that the circulating blood of scabies patients will contain physiologically relevant amounts of SMSB4 or that mite complement inhibitors have a systemic effect in the dermis or in the body.

A clear limitation of the *in vitro* work presented here is that artificial assay systems were utilised and these test conditions mimic the *in vivo* conditions only partially. To provide a clear and detailed picture we tested one single complement inhibitor on its own, which again does not reflect the *in vivo* situation but is the only way to dissect the particular function of this protein. Finally, SMSB4 was produced as a recombinant protein in *E*. *coli* and underwent a lengthy refolding procedure. Hence only a portion of serpin molecules in the preparation was likely biologically active, which may explain why the relatively high concentrations of SMSB4s in a micromolar range were needed to observe a significant complement inhibition in some of the assays presented. Future research should aim to investigate the synergism between scabies mites and pathogenic bacteria in complement inhibition in an *in vivo* setting; potentially suitable animal models have been established [67].

It is intriguing to consider that the collective complement-inhibitory function of multiple mite excretory proteins in combination with complement inhibitors produced by GAS and other bacteria present [14] promotes the survival of bacterial pathogens in the microenvironment of the epidermal burrows produced by the mites. This molecular link between complement inhibition by mite proteins and bacterial survival is a novel aspect of pyoderma pathogenesis that may have important implications for the development of alternative therapies. Improving the treatment and management of scabies requires foremost a better understanding of the interactions between scabies mites, the bacteria subsequently infecting the scabies lesions and the host immune system.

## Supporting information

S1 Fig Testing the deposition of M-, L- and H-ficolins on the cell surface of GAS 88/30. (PDF)
