# Supplementary material for: Complement inhibition by Sarcoptes scabiei protects Streptococcus pyogenes - An in vitro study to unravel the molecular mechanisms behind the poorly understood predilection of S. pyogenes to infect mite-induced skin lesions
Source: PLoS Negl Trop Dis. 2017 Mar 9;11(3):e0005437. doi: 10.1371/journal.pntd.0005437 (PMC5360341; doi:10.1371/journal.pntd.0005437)
Supplement: S1 Fig — (PDF) [file pntd.0005437.s001.pdf]

Testing the deposition of M-, L- and H-ficolins on the cell surface of GAS

Since the Lectin pathway (LP) could be triggered by other lectin pathway PRMs such as M-, L- and H-ficolins, we investigated the deposition of these molecules onto the cell surface of 88/30. None of the ficolins deposited, indicating that the LP may not be important for controlling GAS.

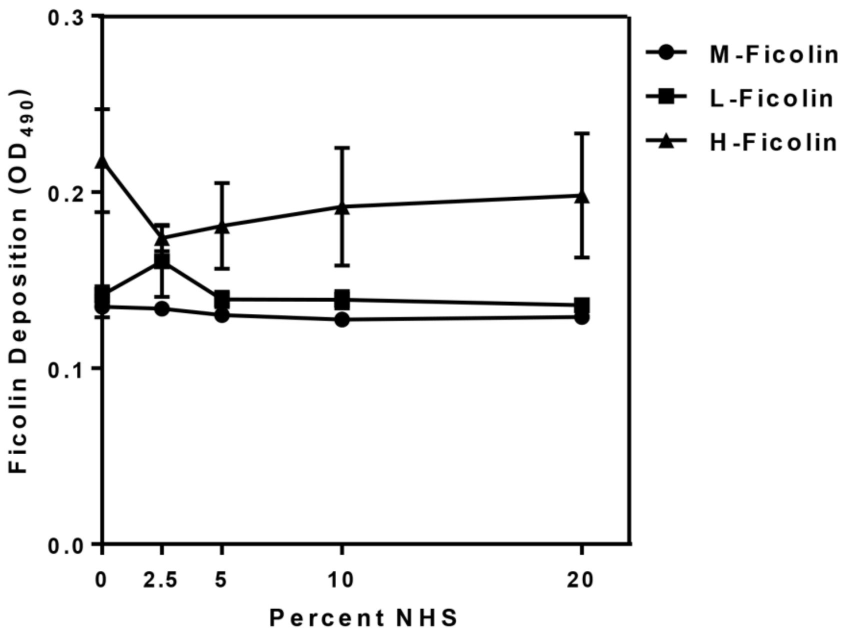

Figure legend:

Maxisorp 96-well plates coated with GAS cells were incubated with increasing concentrations of NHS. Compared to deposition of C1q and FB, no significant Ficolin deposition was detected by ELISA. The statistical significance of differences between samples was estimated using two way ANOVA with Tukey’s multiple comparison test.
